# DNA-Aptamer Raised against Receptor for Advanced Glycation End Products Improves Survival Rate in Septic Mice

**DOI:** 10.1155/2021/9932311

**Published:** 2021-08-07

**Authors:** Yoshinori Koga, Ami Sotokawauchi, Yuichiro Higashimoto, Yuri Nishino, Naoki Hashizume, Tatsuyuki Kakuma, Jun Akiba, Yoshiaki Tanaka, Takanori Matsui, Minoru Yagi, Sho-ichi Yamagishi

**Affiliations:** ^1^Department of Pediatric Surgery, Kurume University School of Medicine, Kurume 830-0011, Japan; ^2^Department of Pathophysiology and Therapeutics of Diabetic Vascular Complications, Kurume University School of Medicine, Kurume 830-0011, Japan; ^3^Department of Chemistry, Kurume University School of Medicine, Kurume 830-0011, Japan; ^4^Biostatistics Center, Kurume University, Kurume 830-0011, Japan; ^5^Department of Pathology, Kurume University School of Medicine, Kurume 830-0011, Japan; ^6^Division of Medical Safety Management, Kurume University Hospital, Kurume 830-0011, Japan; ^7^Division of Diabetes, Metabolism, and Endocrinology, Department of Medicine, Showa University School of Medicine, Tokyo 142-8666, Japan

## Abstract

Despite remarkable scientific advances in the understanding of molecular mechanisms for sepsis, therapeutic options are far from satisfactory. High mobility group box 1 (HMGB1), one of the ligands of receptor for advanced glycation end products (RAGE), is a late mediator of lethality in septic mice. We have recently found that the DNA-aptamer raised against RAGE (RAGE-aptamer) significantly blocks experimental diabetic nephropathy and melanoma growth and metastasis. We examined the effects of RAGE-aptamer on sepsis score, survival rate, and inflammatory and oxidative stress responses in serum, peripheral monocytes, kidneys and livers of lipopolysaccharide- (LPS-) injected mice, and on LPS-exposed THP-1 cells. RAGE-aptamer inhibited the binding of HMGB1 to RAGE *in vitro*. RAGE-aptamer significantly (*P* = 0.002) improved sepsis score at 8 hours after LPS injection and survival rate at 24 hours (*P* < 0.01, 70%) in septic mice compared with LPS+vehicle- or LPS+control-aptamer-treated mice. RAGE-aptamer treatment significantly decreased expression of p-NF-*κ*B p65, an active form of redox-sensitive transcriptional factor, NF-*κ*B and gene or protein expression of TNF-*α*, IL-1*β*, IL-6, and HMGB1 in serum, peripheral monocytes, and kidneys of septic mice in association with the reduction of oxidative stress and improvement of metabolic acidosis, renal and liver damage. LPS-induced oxidative stress, inflammatory reactions, and growth suppression in THP-1 cells were significantly blocked by RAGE-aptamer. Our present study suggests that RAGE-aptamer could attenuate multiple organ damage in LPS-injected septic mice partly by inhibiting the inflammatory reactions via suppression of HMGB1-RAGE interaction.

## 1. Introduction

Sepsis is a public health burden to human health and its care system affects an estimated 30-50 million people all over the world with approximately 5-10 million deaths annually [1–3]. The prevalence of severe sepsis is especially high in critically ill patients with an incidence rate of 270 per 100,000 person-years, and its hospital mortality rate is about 25% [1–3].

Sepsis is defined as maladaptive systemic inflammatory responses to infection, which could lead to potentially life-threatening multiple organ failures [4, 5]. The initial inflammatory response is caused by invading pathogen-derived molecules, such as bacterial lipopolysaccharide (LPS), a component of the outer membrane of Gram-negative bacteria [4–6], which could be involved in a myriad of inflammatory responses in sepsis through the interaction with pattern recognition receptors (PRRs) on immune cells [6]. Despite remarkable scientific advances in the understanding of molecular mechanisms for sepsis, therapeutic options are extremely limited and far from satisfactory, and clinical trials targeting invading bacteria, LPS, or Toll-like receptor-4, one of the main PRRs that could mediate the signaling pathway of LPS, have been disappointing [4–8]. Since most of the failed trials target molecules involved in early inflammatory responses in sepsis, development of a novel therapeutic strategy that specially targets the maladaptive pathway lately activated by sepsis may be urgently needed [9].

High mobility group box 1 (HMGB1), a DNA-binding nonhistone chromosomal protein, is implicated as a late mediator of lethality in septic mice [10, 11]. HMGB1 is released passively from necrotic injured cells or actively by immune cells, such as monocytes, and it works as a putative danger signal involved in pyroptosis and lethality in LPS-injected septic mice via the interaction with receptor for advanced glycation end products (RAGE), the other type of PRR [10–14]. We have recently found that the DNA-aptamer raised against RAGE (RAGE-aptamer) significantly blocks the binding of advanced glycation end products (AGEs), senescent macroprotein derivatives formed at an accelerated rate under diabetes, to RAGE and resultantly attenuates development and progression of experimental diabetic nephropathy, melanoma growth and metastasis, and renal and muscle injuries in animal models of chronic kidney disease [15–20]. However, effects of RAGE-aptamer on lethality and organ damage in septic mice remain unclear. In this study, we examined whether and how RAGE-aptamer could improve survival rate and organ damage in LPS-injected septic mice.

## 2. Materials and Methods

### 2.1. Materials

LPS (*Escherichia coli* O111:B4) (Cat# L2630) and Roswell Park Memorial Institute (RPMI) 1640 medium (Cat# R0883) were obtained from Sigma-Aldrich (St. Louis, MO, USA). The monoclonal antibody raised against 8-hydroxy-2′-deoxyguanosine (8-OHdG) (Cat# MOG-020P) was purchased from Nikken SEIL Corp., Ltd. (Shizuoka, Japan). The anti-HMGB1 monoclonal antibody (Cat# sc-135809, RRID: AB_2264035) and the anti-tumor necrosis factor-*α* (TNF-*α*) monoclonal antibody (Cat# sc-52745, RRID: AB_630345) were purchased from Santa Cruz Biotechnology Inc. (Dallas, TX, USA). The polyclonal antibody raised against interleukin- (IL-) 1*β* (Cat# ab9722) and the anti-kidney injury molecule-1 (KIM-1) polyclonal antibody (Cat# ab47635, RRID: AB_882998) were purchased from Abcam plc. (Cambridge, UK), while the anti-IL-6 polyclonal antibody (Cat# NB600-1131, RRID: AB_10001997) from Novus Biologicals LLC (Centennial, CO, USA).

### 2.2. Preparation of HMGB1, V-Domain of Human RAGE (vRAGE), and RAGE-Aptamer

Recombinant human HMGB1 (residues 1–215) containing a hexahistidine tag was amplified by polymerase chain reaction (PCR) and subcloned into the NdeI and XhoI sites of the pET-21b(+) vector (Novagen, Darmstadt, Germany). The construct was transformed into *Escherichia coli* BL21(DE3) cells, which were grown at 37°C in LB medium containing 50 *μ*g/ml ampicillin, and then induced for 5 hours with 1 mM isopropyl-*β*-D(-)-thiogalactopyranoside at 28°C. Hexahistidine-tagged HMGB1 was purified using Ni Sepharose 6 Fast Flow chromatography (Cat# 17-5318-01, GE Healthcare UK Ltd., Buckinghamshire, UK) followed by anion exchange chromatography. Purity of the sample was confirmed to be >95% using SDS-PAGE and Coomassie Brilliant Blue staining. Preparations of vRAGE (residues 23-121), RAGE-aptamers, and control-aptamer were described previously [15]. Sequences of control-aptamer and three clones of RAGE-aptamers are shown as follows: control-aptamer, 5′-ttcggCctgggGgcggcCagttcGggtccAgtcgcGggag-3′; RAGE-aptamer #1, 5′-ccTgATATggTgTcAccgccgccTTAgTATTggTgTcTAc-3′; RAGE-aptamer #2, 5′-tcTgTTcAggTTggTAcggTggAAggTgTgATTcAcgAgg-3′; RAGE-aptamer #3, 5′-tTccAcTgAgTgccgcggAcTgTTgTTgggAggTggTgTg-3′. Phosphorothioate nucleotides are indicated as capital letters.

### 2.3. Enzyme-Linked Immunosorbent Assay (ELISA)

Effects of RAGE-aptamer or control-aptamer on the binding of HMGB1 to vRAGE were evaluated by ELISA as described previously [15]. Wells precoated with 1 *μ*g vRAGE overnight were incubated with 1 *μ*M HMGB1 and 0.5 *μ*M RAGE-aptamer or control-aptamer for 30 minutes, and then with anti-HMGB1 monoclonal antibody (Cat# 190377, Abcam plc., Cambridge, UK). After 1 hour, horseradish peroxidase-conjugated anti-mouse IgG and 3,3′,5,5′-tetramethylbenzidine were added to the wells, and absorbance at 450 nm was measured.

### 2.4. Preparation of LPS Solution

The lyophilized powder of LPS was dissolved in saline (0.9% NaCl) under sterile conditions followed by heating at 70°C for 1 minute on the day of use.

### 2.5. Animals

Male 8-week-old BALB/c mice (Charles River Laboratories Japan, Inc., Yokohama, Japan) were used. Animal procedures in this study were approved by the Animal Research Committee of Kurume University School of Medicine (Approval number: 2019-230, 2020-167, and 2021-175).

### 2.6. Animal Experiment 1

In order to determine the optimal dose of LPS to cause sepsis in mice, various doses of LPS (15, 20, or 30 *μ*g/g-body weight (BW)) were injected intraperitoneally to the mice. After the injection of LPS, mice were monitored every 2 hours for 24 hours and assessed by the sepsis scoring system according to the method of Shrum et al. [21]. Survival was determined using the Kaplan-Meier method and a log-rank test.

### 2.7. Animal Experiment 2

To examine the effects of RAGE-aptamer on the survival rate of septic mice, control-aptamer (40 pmol/g-BW) or RAGE-aptamer (2, 10, 20, or 40 pmol/g-BW) was administered intraperitoneally in mice 5 minutes after the injection of LPS (20 *μ*g/g-BW). Control mice were given saline twice at 5-minute intervals. Sepsis scores and survival rates were assessed in the same methods as in Animal Experiment 1.

Eight hours after the injection of LPS or first saline (vehicle), blood was collected from the left ventricular of mice. Blood gas measurement was performed immediately after the collection using the GASTAT-710 (Techno Medica Co., Ltd., Kanagawa, Japan). Blood lactate was measured using a Lactate Pro2 LT-1730 (ARKRAY, Inc., Kyoto, Japan). Complete blood counts were measured using a VetScan HM5 (Abaxis, Inc., Union City, CA, USA). Serum electrolytes, alanine transaminase (ALT), aspartate transaminase (AST), blood urea nitrogen (BUN), creatinine, and lactate dehydrogenase (LDH) were analyzed using a FUJI DRI-CHEM 4000 V chemistry analyzer (Fujifilm Ltd., Tokyo, Japan). Kidneys and livers were obtained from mice at 0.5, 2, 4, 6, or 8 hours after the injection of LPS or first saline for morphological analyses, and urine was collected for measurements of 8-OHdG and KIM-1.

### 2.8. Immunostaining and Morphological Analysis

The specimen of kidneys and livers were fixed with 4% paraformaldehyde, embedded in paraffin, sectioned at 4 *μ*m intervals and mounted on glass slides. The sections were left in 0.3% hydrogen peroxide for 30 minutes to block endogenous peroxidase activity and incubated overnight at 4°C with primary antibodies, and the reactions were visualized with a Histofine Simple Stain Mouse MAX PO (MULTI) kit (Cat# 424152, Nichirei Bioscience Inc., Tokyo, Japan) as described before [19]. Ten images were captured per section from individual mice in each group. Immunohistoreactivity fields in each sample were measured by cellSens version 1.14 software (Olympus Corp., Tokyo, Japan). Three-micrometer paraffin sections were stained with hematoxylin and eosin (HE) for light microscopic analysis. Severity grade for tubular degeneration/necrosis score in the HE-stained kidney specimen was assessed according to the method of Bellinger et al. [22]. Hepatic necrosis area accompanied with inflammatory cell aggregation in the HE-stained liver specimen was calculated with cellSens version 1.14 software.

### 2.9. Isolation of Peripheral Blood Mononuclear Cells (PBMCs)

Heparinized whole blood samples were collected from mice, and then centrifuged at 1,200 × g for 10 minutes. PBMCs were isolated using SepMate-15 tubes (Cat# 15420, STEMCELL Technologies Inc., Cambridge, UK) and Lymphoprep (Cat# 1114545, Axis-Shield Diagnostics Ltd., Dundee, UK).

### 2.10. Cell Experiments

Human THP-1 monocytic leukemia cells (American Type Culture Collection, Manassas, VA, USA) were routinely cultured in RPMI-1640 medium supplemented with 5% fetal bovine serum at 37°C. In all experiments, THP-1 cells were seeded into plates at a density of 1 × 10^6^ cells/ml and treated with 100 ng/ml LPS or saline (control) in the presence or absence of RAGE-aptamer or control-aptamer. Intracellular reactive oxygen species (ROS) production in THP-1 cells was measured with a fluorescent probe, 5-(and-6)-carboxy-2′,7′-difluorodihydrofluorescein diacetate (carboxy-H_2_DFFDA) (Cat# C13293, Thermo Fisher Scientific, San Jose, CA, USA) as described previously [15]. Viable cell number of THP-1 cells was determined by MTT- (3-(4,5-dimethylthiazol-2-yl)-2,5-diphenyltetrazolium bromide-) based colorimetric assay according to the supplier's instructions (Cat# CBA-252, Cell Biolabs, Inc., San Diego, CA, USA).

### 2.11. Measurements of Nuclear Factor- (NF-) *κ*B, Cytokines, and Urinary 8-OHdG and KIM-1

Total NF-*κ*B p65 and phosphorylated NF-*κ*B p65 at Serine536 (p-NF-*κ*B p65) in PBMCs and THP-1 cells were analyzed with the NF-*κ*B p65 (pS536+total) ELISA Kit (Cat# ab176663, Abcam plc., Cambridge, UK). TNF-*α*, IL-1*β*, and IL-6 in the serum and cell supernatants and urinary KIM-1 were determined with ELISA kits (Cat# MTA00B, MLB00C, M6000B, DTA00D, DLB50, D6050, and MKM100, R&D Systems, Minneapolis, MN, USA, respectively). HMGB1 in the serum and cell supernatant were measured with an ELISA kit purchased from Shino-Test Corp. (Cat# 326054329, Tokyo, Japan). Urinary levels of 8-OHdG were measured with an ELISA kit (Cat# KOG-HS10/E, Nikken SEIL Corp., Ltd.).

### 2.12. Reverse Transcription-PCR (RT-PCR)

Total RNA was extracted from PBMCs and kidneys of mice and cultured THP-1 cells with the NucleoSpin RNA Plus kit (Cat# U0984C, Takara Bio Inc., Shiga, Japan) according to the manufacturer's instructions. Quantitative real-time RT-PCR was performed using Assay-on-Demand and TaqMan 5 ′ fluorogenic nuclease chemistry (Life Technologies Japan Ltd., Tokyo, Japan) according to the supplier's recommendation. IDs of primers for mouse p22phox, NADPH oxidase 2 (Nox2, also known as gp91phox), p47phox, p67phox, TNF-*α*, IL-1*β*, IL-6, HMGB1, KIM-1, and *β*-actin genes were Mm00514478_m1, Mm01287743_m1, Mm00447921_m1, Mm00726636_s1, Mm00443258_m1, Mm01336189_m1, Mm00446190_m1, Mm00849805_gH, Mm00506686_m1, and Mm00607939_s1, respectively. Identifiers of primers for human p22phox, Nox2, p47phox, p67phox, TNF-*α*, IL-1*β*, IL-6, HMGB1, and *β*-actin genes were Hs00609145_m1, Hs00166163_m1, Hs00417167_m1, Hs1084940_m1, Hs00174128_m1, Hs00174097_m1, Hs00985639_m1, Hs01923466_g1, and Hs99999903_m1, respectively. Data were normalized by the intensity of *β*-actin-derived signals and then related to the value obtained with saline-injected control mice or saline-treated control cells.

### 2.13. Measurement of NADPH Oxidase Activity

Renal NADPH oxidase activity and cultured THP-1 cells were measured by a luminescence assay as described previously [15].

### 2.14. Statistical Analysis

**Figure 1:**
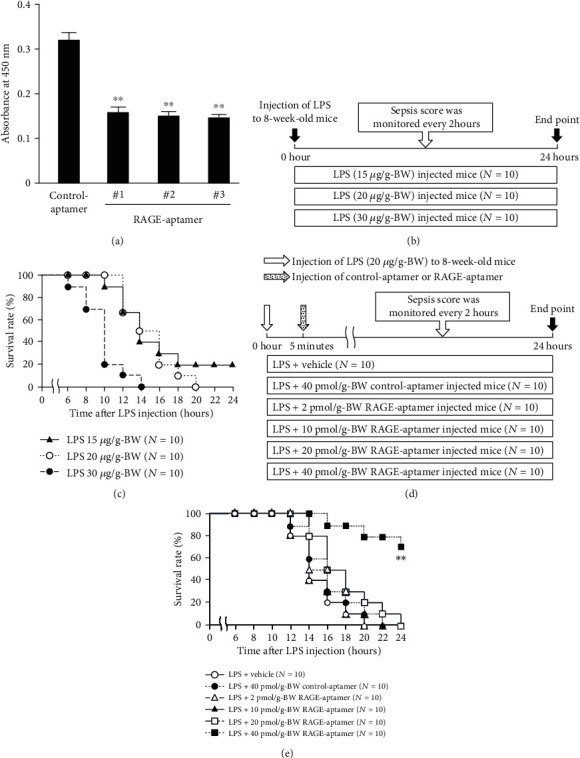
(a) ELISA for the binding of HMGB1 to vRAGE in the presence of control-aptamer or RAGE-aptamers. *N* = 4 per group. ^∗∗^*P* < 0.01 compared with control-aptamer. (b) Experimental design 1. (c) The Kaplan-Meier survival analysis of mice injected with the indicated doses of LPS (each *N* = 10). (d) Experimental design 2. (e) The Kaplan-Meier survival analysis of mice injected with LPS (20 *μ*g/g-BW) plus vehicle (saline) in the presence or absence of the indicated doses of RAGE-aptamer or 40 pmol/g-BW of control-aptamer. *N* = 10 per group. ^∗∗^*P* < 0.01 compared with control-aptamer.

**Figure 2:**
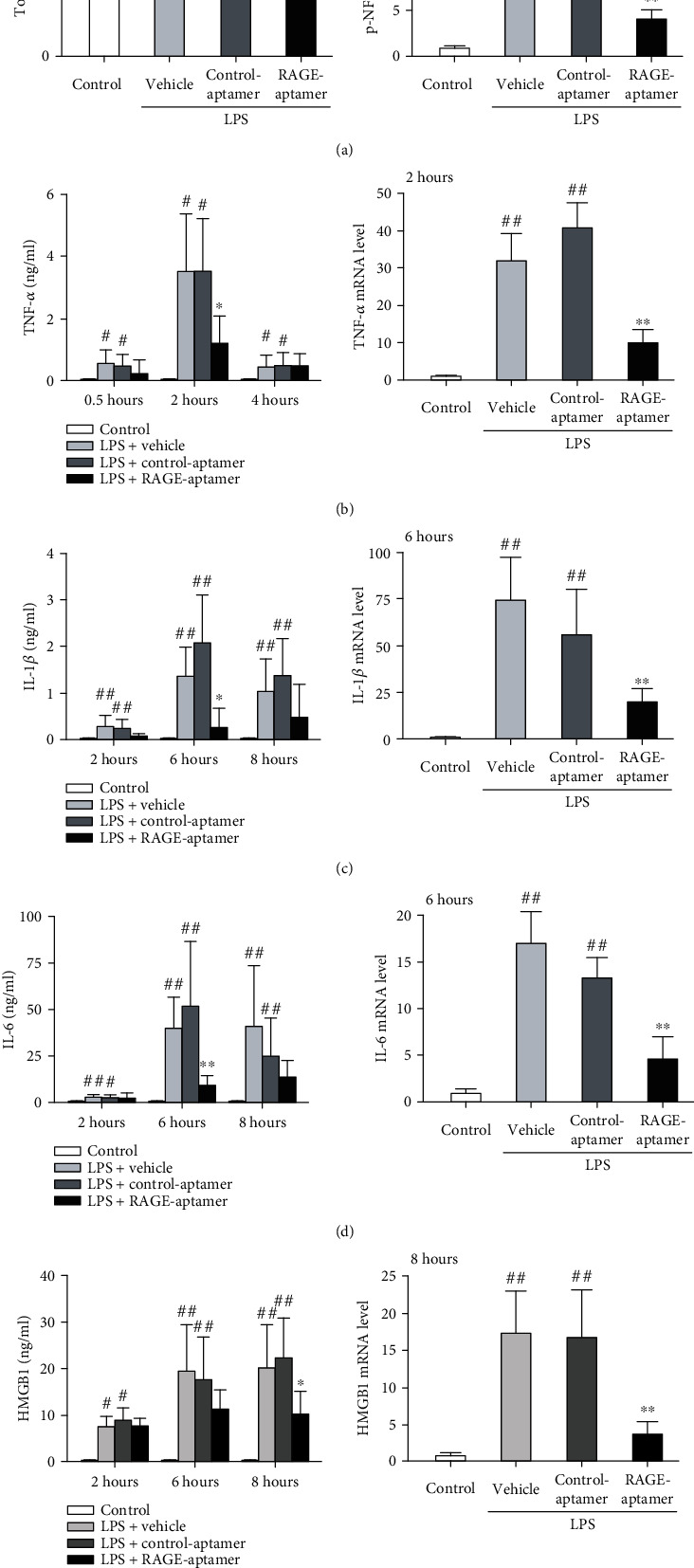
NF-*κ*B p65, gene and protein expression of cytokines in serum and PBMCs of mice injected with saline (control), LPS (20 *μ*g/g-BW) plus vehicle (positive control), LPS (20 *μ*g/g-BW) plus 40 pmol/g-BW of control-aptamer, or LPS (20 *μ*g/g-BW) plus 40 pmol/g-BW of RAGE-aptamer. (a) Total NF-*κ*B p65 (left panel) and p-NF-*κ*B p65 levels (right panel) in PBMCs. (b–e) Each left panel shows serum levels of TNF-*α* (b), IL-1*β* (c), IL-6 (d), and HMGB1 (e). *N* = 7 per group. Each right panel shows gene expression levels of cytokines in PBMCs. *N* = 5 per group. ^#^*P* < 0.05 and ^##^*P* < 0.01 compared with control, respectively. ^∗^*P* < 0.05 and ^∗∗^*P* < 0.01 compared with LPS plus control-aptamer-treated mice, respectively.

**Figure 3:**
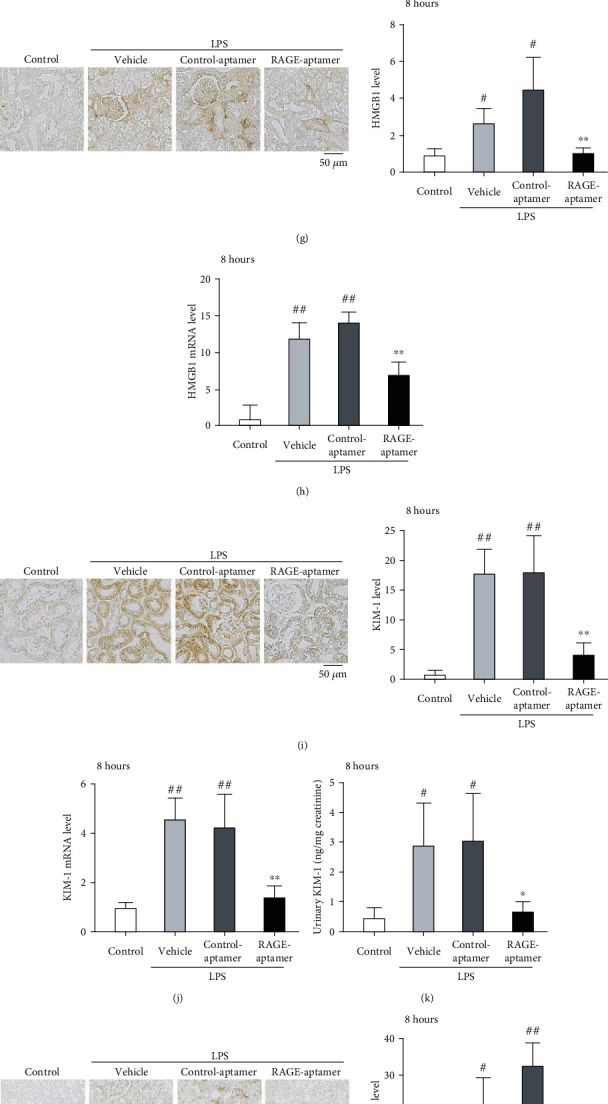
Effects of RAGE-aptamer on kidney and liver damage in septic mice. (a, c, e, g, and i) Each left panel shows the representative immunostainings of TNF-*α* (a), IL-1*β* (c), IL-6 (e), HMGB1 (g), and KIM-1 (i). Each right panel shows the quantitative data. (b, d, f, h, and j) mRNA levels of TNF-*α* (b), IL-1*β* (d), IL-6 (f), HMGB1 (h), and KIM-1 (j) in the kidneys. (k) Urinary KIM-1 levels. (l) Left panels show the representative immunostainings of 8-OHdG. Right panel shows the quantitative data. (m) Urinary 8-OHdG levels. (n) NADPH oxidase-derived superoxide generation. (o–r) mRNA levels of p22phox (o), Nox2 (p), p47phox (q), and p67phox (r) in the kidneys. (s) Left panels show the representative HE staining of kidneys. Right panel shows the scores for tubular degeneration/necrosis. (t) Left panels show the representative HE staining of livers. Right panel shows the quantitative data of necrosis area. *N* = 5 per group. ^#^*P* < 0.05 and ^##^*P* < 0.01 compared with control, respectively. ^∗^*P* < 0.05 and ^∗∗^*P* < 0.01 compared with LPS plus control-aptamer-treated mice, respectively. ^††^*P* < 0.01 compared with LPS+vehicle-treated mice.

**Figure 4:**
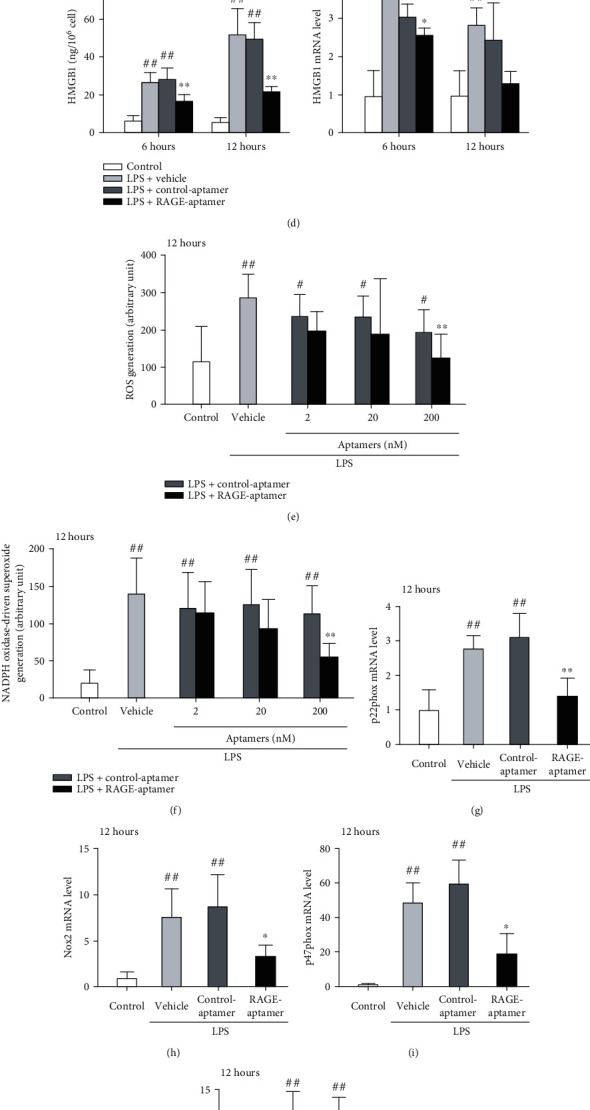
Effects of RAGE-aptamer on LPS-exposed THP-1 cells. (a–d) Each left panel shows the protein levels of TNF-*α* (a), IL-1*β* (b), IL-6 (c), and HMGB1 (d) produced by THP-1 cells. Each right panel shows the mRNA levels of cytokines in THP-1 cells. (e) ROS generation. (f) NADPH oxidase-derived superoxide generation. (g–j) mRNA levels of p22phox (g), Nox2 (h), p47phox (i), and p67phox (j). (k) Total NF-*κ*B p65 (left panel) and p-NF-*κ*B p65 levels (right panel) in THP-1 cells. (l) Viable cell number of THP-1 cells. *N* = 5 per group. ^#^*P* < 0.05 and ^##^*P* < 0.01 compared with control, respectively. ^∗^*P* < 0.05 and ^∗∗^*P* < 0.01 compared with LPS+control-aptamer-treated cells, respectively. ^†^*P* < 0.05 and ^††^*P* < 0.01 compared with LPS+vehicle-treated cells, respectively.

All values were expressed as mean ± standard deviation. Survival curve by the Kaplan-Meier method was evaluated by log-rank test (Figures 1(c) and 1(e)). Comparisons of LPS-induced sepsis scores between control-aptamer-treated and RAGE-aptamer-treated mice were performed in the proportional hazard model by including sepsis score and group interaction terms (Table 1). Statistical comparisons were performed using ANOVA followed by the Steel-Dwass test (Figures 2(b) (ELISA 0.5, 2, and 4 hours), Figure 2(c) (ELISA 2, 6, and 8 hours), Figure 2(d) (ELISA 8 hours), Figure 2(e) (ELISA 2 hours), Figures 3(k), 3(m), 3(n), 4(a) (ELISA 6 hours and mRNA 2 hours), Figure 4(b) (ELISA 12 hours and mRNA 6 hours), Figure 4(c) (ELISA 6 and 12 hours), Figure 4(d) (mRNA 12 hours), Figures 4(e), 4(f), 4(h), 4(i), and 4(k) (p-NF-*κ*B p65), and Table 2 (PaO_2_, pH, lactate, and potassium)). The rest of the data were analyzed by the Tukey-Kramer test. *P* < 0.05 was considered significant. All statistical analyses were performed with JMP Pro version 13.0 (SAS Institute Inc., Cary, NC, USA).

## 3. Results

### 3.1. Effects of RAGE-Aptamers on HMGB1-vRAGE Interaction

We have already found that RAGE-aptamer clones #1, #2, and #3 inhibit the binding of AGEs to vRAGE [15]. Therefore, we examined the effects of these aptamers on HMGB1-vRAGE interaction. As shown in Figure 1(a), all three clones significantly inhibited the binding of HMGB1 to vRAGE. HMGB1 was bound to RAGE with a dissociation constant (*K*_d_) of 48.3 nM. In the presence of RAGE-aptamer #1, the *K*_d_ value exhibited two orders of magnitude higher level [16]. Since we have reported that RAGE-aptamer clone #1 did not exert nonspecific toxic effects in both cell culture and animal model systems [15, 19], we used RAGE-aptamer clone #1 for the following experiments.

### 3.2. Survival Rate in Animal Experiment 1

The Kaplan-Meier analysis revealed that LPS injection dose-dependently decreased the survival rate of septic mice (Figures 1(b)–1(c)); all mice were dead by 14 hours after 30 *μ*g/g-BW LPS injection, while mice received 20 *μ*g/g-BW LPS died between 12 and 20 hours after the administration (Figure 1(c)).

We next evaluated the effects of sepsis score at 8 hours on the survival time among three doses of LPS-injected groups by the proportional hazard model. When effects of the sepsis score was evaluated by the hazard ratio within each dose group, hazard ratios (HR) for 15 *μ*g-BW LPS, 20 *μ*g-BW LPS, and 30 *μ*g-BW LPS were 1.52 (*P* = 0.039), 2.60 (*P* = 0.012), and 0.95 (*P* = 0.970), respectively (Table 1). Since *P* value of HR was the smallest in 20 *μ*g-BW LPS-treated mice, we chose the condition in the following experiments.

### 3.3. Survival Rate in Animal Experiment 2

We examined the effects of the indicated doses of RAGE-aptamer on the survival rate of septic mice (Figure 1(d)). As shown in Figure 1(e), compared with vehicle or 40 pmol/g-BW control-aptamer, treatment with the same concentration of RAGE-aptamer significantly improved the survival rate of 20 *μ*g/g-BW LPS-injected septic mice, while median survival time and survival rate at 24 hours after LPS injection in the control-aptamer-treated group were 16 hours and 0%, respectively; the latter was drastically extended to 70% in RAGE-aptamer-treated mice. Those in vehicle-treated septic mice were 14 hours and 0%, respectively.

Then, we compared the effects of sepsis score measured at 8 hours after LPS injection on the survival time between the 40 pmol/g-BW RAGE-aptamer- and 40 pmol/g-BW control-aptamer-treated groups by the proportional hazard model with sepsis score and group interaction terms. As shown in Table 3, the likelihood ratio test indicated a significant interaction (likelihood ratio = 5.8, degrees of freedom = 1, *P* = 0.016); there was a significant difference in sepsis score effects between the two groups. Based on the estimate of hazard ratio, high sepsis score was associated with the increased risk of death (HR = 2.6, *P* = 0.005), and average sepsis score in the 40 pmol/g-BW RAGE-aptamer-treated group was significantly lower (*P* = 0.002) than that in the 40 pmol/g-BW control-aptamer-treated group (6.6 ± 1.6 vs. 10.3 ± 2.7).

### 3.4. Effects of RAGE-Aptamer on Blood Gas, Electrolytes, and Biochemical Parameters

As shown in Table 2, compared with control nonseptic mice, PaO_2_, lactate, anion gap, red blood cells, hemoglobin, BUN, creatinine, AST, and ALT were significantly increased in vehicle-treated- or control-aptamer-treated septic mice, while HCO_3_^−^, pH, base excess, white blood cells, and platelets were decreased. RAGE-aptamer treatment significantly restored HCO_3_^−^, pH, base excess, and platelets and simultaneously decreased lactate, anion gap, BUN, creatinine, AST, and ALT levels. There was a significant difference of anion gap between vehicle-treated- and control-aptamer-treated septic mice.

### 3.5. Effects of RAGE-Aptamer on Cytokine Expression in Septic Mice

Serum and PBMCs were obtained from septic mice at the indicated time periods after LPS or first saline injection. As shown in Figure 2, p-NF-*κ*B p65, TNF-*α*, IL-1*β*, IL-6, and HMGB1 mRNA levels in PBMCs isolated from vehicle-treated- or control-aptamer-treated septic mice were significantly increased compared with control nonseptic mice, which were associated with the increases in serum levels of TNF-*α*, IL-1*β*, IL-6, and HMGB1 (Figure 2). RAGE-aptamer treatment significantly reduced these inflammatory parameters in both PBMCs and serum obtained from septic mice.

### 3.6. Effects of RAGE-Aptamer on Renal and Liver Injuries in Septic Mice

Gene and protein expression levels of TNF-*α*, IL-1*β*, IL-6, HMGB1, and KIM-1, a marker of renal damage, and urinary KIM-1 levels were significantly increased in vehicle-treated- or control-aptamer-treated septic mice (Figures 3(a)–3(k)). Treatment of RAGE-aptamer significantly decreased all of these parameters compared with LPS plus control-aptamer-treated mice (Figures 3(a)–3(k)). Furthermore, renal 8-OHdG, a marker of oxidative stress, NADPH oxidase-derived superoxide generation, and gene expression of components of NADPH oxidase except for p67phox were significantly increased in vehicle-treated- or control-aptamer-treated septic mice in association with the elevation of urinary 8-OHdG levels (Figures 3(l)–3(r)). RAGE-aptamer treatment significantly reduced the expression levels of these oxidative stress markers in the kidneys of septic mice compared with control-aptamer-treated septic mice (Figures 3(l)–3(r)). HE staining revealed that renal tubular degeneration and hepatic necrosis area accompanied with inflammatory cell aggregation were increased in vehicle-treated- or control-aptamer-treated septic mice, both of which were ameliorated by RAGE-aptamer (Figures 3(s)–3(t)). There was a significant difference of Nox2 mRNA levels in the kidneys between vehicle-treated- and control-aptamer-treated septic mice.

### 3.7. Effects of RAGE-Aptamer on LPS-Exposed THP-1 Cells

LPS significantly increased gene expression in, and protein production of TNF-*α*, IL-1*β*, IL-6, and HMGB1 by, vehicle- or control-aptamer-treated THP-1 cells, which were attenuated by RAGE-aptamer treatment (Figures 4(a)–4(d)). Moreover, RAGE-aptamer at 200 nM significantly reduced the LPS-induced ROS generation, NADPH oxidase-driven superoxide production, gene expression of components of NADPH oxidase, and p-NF-*κ*B p65 in THP-1 cells (Figures 4(e)–4(k)). LPS significantly reduced the viable cell number of THP-1 cells, which was restored by RAGE-aptamer at 200 nM (Figure 4(l)). There were no significant differences in these parameters, except for IL-6 at 12 hours and HMGB1 mRNA levels at 6 hours between LPS+vehicle-treated cells and LPS+control-aptamer-treated cells.

## 4. Discussion

LPS, also known as endotoxin, has been shown to play a central role in the pathogenesis of Gram-negative bacteria-induced sepsis [6, 23]. In order to investigate the efficacy of RAGE-aptamer on the survival rate of septic mice, we chose the condition of intraperitoneal injection of 20 *μ*g/g-BW LPS because the effect of sepsis score measured at 8 hours on survival time was the largest after this dose of LPS injection. In the present study, we found for the first time that RAGE-aptamer significantly inhibited the binding of HMGB1 to vRAGE *in vitro* and that intraperitoneal administration of 40 pmol/g-BW RAGE-aptamer improved the sepsis score measured at 8 hours after LPS injection and survival rate in LPS-injected septic mice. Furthermore, RAGE-aptamer treatment significantly attenuated oxidative stress and inflammatory reactions, including HMGB1 expression in serum, PBMCs, and kidneys of LPS-injected septic mice in association with the improvement of renal and liver injuries and metabolic acidosis. We also found here that RAGE-aptamer inhibited the LPS-induced oxidative stress generation in, and secretion of cytokines and HMGB1 by, THP-1 cells. Therefore, the present study suggests that RAGE-aptamer could attenuate multiple organ damage in LPS-injected septic mice partly by inhibiting the inflammatory reactions via suppression of HMGB1-RAGE interaction.

Although the pathological role of RAGE in sepsis has been evaluated in several kinds of animal models, there is some controversy about the effects of RAGE gene deficiency on sepsis in experimental models [24–30]. A couple of papers have reported that RAGE-deficient mice exhibited a significant protection against inflammation and lethality due to polymicrobial sepsis caused by cecal ligation and puncture or intranasal inoculation with *Streptococcus pneumoniae* [25–27]. However, others have shown that survival rate is not improved, or rather deteriorated, in septic mice with intratracheal instillation of LPS or *Escherichia coli*, intravenous injection of *Streptococcus pneumoniae*, or intranasal inoculation of LPS or *Klebsiella pneumoniae* [28–30]. In addition, as far as we know, there are only two papers to show that administration of anti-RAGE antibody significantly improves the survival rate in septic mice of cecal ligation and puncture model or intratracheal infection with *Streptococcus pneumoniae* [25, 31]. In this study, we showed first that RAGE-aptamer administration just after LPS injection not only ameliorated the sepsis score and survival rate, but also attenuated critical organ damage in septic mice, thus suggesting that RAGE is a therapeutic target for sepsis.

Nucleic acid aptamers are short single-stranded oligonucleotides that are able to bind to various kinds of target proteins like antibodies, which are selected by systemic evolution of ligands by exponential enrichment [32, 33]. Compared with neutralizing antibodies, aptamers are more easily prepared and could more efficiently penetrate into various tissues with less immunogenicity and more thermal stability [32, 33]. Its advantages over antibodies for blocking target proteins make nucleic acid aptamers a very attractive tool for *in vivo*-therapeutic application. Indeed, pegaptanib (Macugen), an RNA-aptamer raised against vascular endothelial growth factor_165_, has already been approved by the U.S. Food and Drug Administration for the treatment of the wet type of age-related macular degeneration, while several types of aptamers directed against coagulation systems have undergone clinical trials [33, 34]. In addition to these things, we found here that RAGE-aptamer treatment after the LPS injection exhibited protective effects on organ damage and death in septic mice without adverse side effects. Given that HMGB1 is a late lethality mediator in sepsis [10, 11], our present findings may support the clinical feasibility of RAGE-aptamer for treatment of severe sepsis.

In the present study, RAGE-aptamer treatment significantly decreased expression of p-NF-*κ*B p65, an active form of redox-sensitive transcriptional factor, NF-*κ*B [35, 36], and gene expression of TNF-*α*, IL-1*β*, and IL-6, and subsequently reduced HMGB1 mRNA levels in PBMCs isolated from septic mice. Furthermore, serum levels of these cytokines and HMGB1 were increased in LPS-injected septic mice, all of which were significantly blocked by RAGE-aptamer. HMGB1 has been shown to stimulate NADPH oxidase-induced ROS generation and subsequently induce NF-*κ*B activation in numerous types of cells through the interaction with PRRs, including RAGE, which could lead to evoke inflammatory reactions [37–40]. Moreover, oxidative stress and inflammatory reactions have been reported to stimulate HMGB1 release and activity [37–40]. These observations suggest a positive feedback loop between HMGB1-RAGE-induced ROS generation as well as inflammatory reactions and HMGB1 expression in septic mice. RAGE-aptamer could break the crosstalk between HMGB1-RAGE axis and ROS, thereby improving the sepsis score and survival rate in LPS-injected septic mice.

In this study, we also found that treatment with RAGE-aptamer significantly attenuated gene and protein expression of TNF-*α*, IL-1*β*, and IL-6, and HMGB1 in the kidneys of septic mice at 2-8 hours after LPS injection. Subsequently, 8-OHdG, a marker of oxidative stress, NADPH oxidase activity, and gene expression of components of NADPH oxidase except for p67phox were increased in the kidneys of septic mice in association of elevation of urinary 8-OHdG and KIM-1, one of the markers of proximal tubular damage and acute kidney injury [41], renal dysfunction as evaluated by BUN and creatinine levels, metabolic acidosis, and renal tubular degeneration, all of which were inhibited by RAGE-aptamer. Therefore, RAGE-aptamer could protect against renal damage in septic mice partly by suppressing the vicious cycle among HMGB1-RAGE axis, ROS generation, and inflammatory reactions in the kidneys. In addition, we found here that RAGE-aptamer significantly attenuated liver injury in septic mice, which were indicated by a decrease in serum levels of ALT and AST and amelioration of hepatic necrosis in HE staining.

We also found in the present study that LPS-induced increases in TNF-*α*, IL-1*β*, and IL-6, and HMGB1 gene and protein expression, ROS generation, activity and gene expression of components of NADPH oxidase, and p-NF-*κ*B p65 in THP-1 cells were significantly blocked by RAGE-aptamer. Moreover, RAGE-aptamer inhibited the LPS-induced decrease in viable cell number of THP-1 cells. These *in vitro*-findings further support the concept that a maladaptive feedforward loop between the HMGB1-RAGE axis and inflammatory reactions in immune cells could partly contribute to organ damage and death in LPS-induced sepsis and that blockade of the system by RAGE-aptamer may be a therapeutic target for the devastating disorder. Pyroptosis, an inflammatory form of programed cell death, has been shown to be involved in LPS-HMGB1-induced sepsis [13]. Since inflammatory reactions and reduced viable cell number of LPS-exposed THP-1 cells were simultaneously prevented by RAGE-aptamer, our present study suggests that RAGE-aptamer may inhibit the pyroptosis of immune cells in LPS-induced septic mice.

There are a couple of limitations in the present study. It remains unclear whether other RAGE ligands other than HMGB1 could play a role in our model. It would be helpful to investigate the pathological role of other RAGE ligands other than HMGB1, such as S100 proteins in LPS-injected septic mice [42]. Furthermore, since the protein and mRNA levels of IL-6 treated with RAGE-aptamer were still rising from 6 to 12 hours, it would be also interesting to examine the effects of RAGE-aptamer on IL-6 expression on time points post 12 hours.

## 5. Conclusion

Our present study suggests that RAGE-aptamer could attenuate multiple organ damage in LPS-injected septic mice partly by inhibiting the inflammatory reactions via suppression of HMGB1-RAGE interaction.

## Figures and Tables

**Table 1:** Effects of sepsis score at 8 hours on survival time of mice.

	8-week-old mice		
LPS dosage	15 *μ*g/g-BW	20 *μ*g/g-BW	30 *μ*g/g-BW
Number	10	10	10
BW (g)	26.8 (0.9)	26.6 (0.9)	25.7 (1.4)
Sepsis score	10.0 (1.8)	9.5 (2.0)	17.4 (1.6)
HR	1.52	2.6	0.95
95% CI on HR	1.02, 2.52	1.37, 6.31	0.06, 13.92
*P* value	0.039	0.012	0.970

Data are presented as mean (standard deviation). LPS: lipopolysaccharide; BW: body weight; HR: hazard ratio; CI: confidence interval.

**Table 2:** Blood gas, electrolytes, and biochemical parameters.

Characteristics	8-week-old mice			
	Control	LPS+vehicle	LPS+control-aptamer	LPS+RAGE-aptamer
Number	9	9	9	9
PaO_2_ (Torr)	83.7 (10.5)	154.9 (34.3)^##^	145.8 (32.6)^##^	115.0 (35.7)
PaCO_2_ (Torr)	31.9 (3.5)	34.1 (8.9)	34.4 (6.3)	35.6 (6.9)
HCO_3_^−^ (mmol/l)	17.6 (0.9)	10.3 (1.7)^##^	10.7 (1.4)^##^	12.4 (0.9)^∗^
pH	7.32 (0.02)	7.06 (0.05)^##^	7.08 (0.05)^##^	7.14 (0.03)^∗^
Base excess (mEq/l)	-8.9 (1.2)	-19.9 (3.2)^##^	-19.3 (2.5)^##^	-16.2 (1.5)^∗^
Lactate (mmol/l)	1.7 (0.2)	3.6 (0.8)^##^	3.6 (0.9)^##^	2.2 (0.4)^∗∗^
Anion gap	21.7 (3.0)	34.1 (2.3)^##^	29.6 (2.2)^##,†^	24.2 (2.9)^∗∗^
Sodium (mEq/l)	149.2 (2.3)	152.0 (5.8)	150.6 (3.0)	149.6 (1.8)
Potassium (mEq/l)	4.7 (0.5)	6.1 (0.8)	6.0 (0.9)	6.4 (1.2)
Chloride (mEq/l)	109.9 (3.5)	107.6 (4.5)	110.2 (2.6)	112.9 (3.5)
Number	7	7	7	7
RBC (×10^9^/l)	10.4 (0.4)	11.5 (0.4)^##^	11.2 (1.0)^##^	11.0 (0.3)
WBC (×10^9^/l)	4.4 (1.6)	2.3 (0.6)^##^	2.1 (0.8)^##^	1.7 (0.6)
Hemoglobin (mg/dl)	15.4 (1.0)	17.4 (0.7)^##^	17.3 (1.4)^##^	16.4 (0.4)
Platelets (10^6^/mm^3^)	39.0 (5.3)	20.7 (2.1)^##^	20.0 (4.5)^##^	27.7 (5.8)^∗^
BUN (mg/dl)	23.1 (1.3)	60.2 (5.9)^##^	65.7 (6.3)^##^	33.9 (13.3)^∗∗^
Creatinine (mg/dl)	0.26 (0.06)	0.70 (0.23)^#^	0.73 (0.27)^#^	0.32 (0.11)^∗∗^
AST (U/l)	38.1 (4.0)	165.6 (59.8)^##^	180.9 (55.1)^##^	89.7 (26.6)^∗∗^
ALT (U/l)	58.7 (18.8)	123.0 (10.6)^##^	143.0 (25.2)^##^	77.1 (29.7)^∗∗^
LDH (U/l)	1201.7 (561.7)	1583.4 (1028.9)	1249.1 (326.6)	1088.0 (398.4)

Data are presented as mean (SD). ^#^*P* < 0.05 and ^##^*P* < 0.01 compared with control mice, respectively. ^∗^*P* < 0.05 and ^∗∗^*P* < 0.01 compared with mice that received LPS+control-aptamer, respectively. ^†^*P* < 0.05 compared with mice that received LPS+vehicle. RBC: red blood cells; WBC: white blood cells; BUN: blood urea nitrogen; AST: aspartate aminotransferase; ALT: alanine aminotransferase; LDH: lactate dehydrogenase.

**Table 3:** Proportional hazard model containing sepsis score and group interaction terms.

Parameters	LRT	*P* value	df
Group	3.0	0.083	1
Sepsis score	7.9	0.005	1
Group∗sepsis score	5.8	0.016	1

LRT: likelihood ratio chi-squared test; df: degrees of freedom.

## Data Availability

The datasets used and analyzed during the current study are available from the corresponding author on reasonable request.
